# perfDSA: Automatic Perfusion Imaging in Cerebral Digital Subtraction Angiography

**DOI:** 10.1007/s11548-025-03359-4

**Published:** 2025-04-24

**Authors:** Ruisheng Su, P. Matthijs van der Sluijs, Flavius-Gabriel Marc, Frank te Nijenhuis, Sandra A. P. Cornelissen, Bob Roozenbeek, Wim H. van Zwam, Aad van der Lugt, Danny Ruijters, Josien Pluim, Theo van Walsum

**Affiliations:** 1 Department of Biomedical Engineering, Eindhoven University of Technology, Eindhoven, The Netherlands; 2 Department of Radiology & Nuclear Medicine, Erasmus MC, University Medical Center Rotterdam, Rotterdam, The Netherlands; 3 Department of Radiology & Nuclear Medicine, Maastricht University Medical Center, Maastricht, The Netherlands; 4 Interventional X-Ray (iXR), Philips Healthcare, Best, The Netherlands

**Keywords:** Digital subtraction angiography, Deep learning, Vessel segmentation, Cerebral blood flow, Perfusion, Cerebrovascular disease

## Abstract

**Purpose:**

Cerebral digital subtraction angiography (DSA) is a standard imaging technique in image-guided interventions for visualizing cerebral blood flow and therapeutic guidance thanks to its high spatio-temporal resolution. To date, cerebral perfusion characteristics in DSA are primarily assessed visually by interventionists, which is time-consuming, error-prone, and subjective. To facilitate fast and reproducible assessment of cerebral perfusion, this work aims to develop and validate a fully automatic and quantitative framework for perfusion DSA.

**Methods:**

We put forward a framework, perfDSA, that automatically generates deconvolution-based perfusion parametric images from cerebral DSA. It automatically extracts the arterial input function from the supraclinoid internal carotid artery (ICA) and computes deconvolution-based perfusion parametric images including cerebral blood volume (CBV), cerebral blood flow (CBF), mean transit time (MTT), and Tmax.

**Results:**

On a DSA dataset with 1006 patients from the multicenter MR CLEAN registry, the proposed perfDSA achieves a Dice of 0.73(±0.21) in segmenting the supraclinoid ICA, resulting in high accuracy of arterial input function (AIF) curves similar to manual extraction. Moreover, some extracted perfusion images show statistically significant associations (*P*=2.62e$-$5) with favorable functional outcomes in stroke patients.

**Conclusion:**

The proposed perfDSA framework promises to aid therapeutic decision-making in cerebrovascular interventions and facilitate discoveries of novel quantitative biomarkers in clinical practice. The code is available at https://github.com/RuishengSu/perfDSA.

**Supplementary Information:**

The online version contains supplementary material available at 10.1007/s11548-025-03359-4.

## Introduction

Cerebral X-ray digital subtraction angiography (DSA) is currently the gold standard in interventional radiology for blood flow visualization and therapeutic guidance in endovascular treatments for neurovascular conditions such as stroke and intracranial aneurysysms [1]. This imaging modality yields a contrast-enhanced time-resolved 2D image series in anteroposterior and lateral projection obtained by subtracting the pre-contrast frame from post-contrast ones. This subtraction technique effectively removes the visual interference of static soft tissue and bone, highlighting only the contrast-filled vessels [2]. To date, DSA provides real-time imaging with unparalleled spatial and temporal resolution, assisting interventionists to appreciate the intricate structure and flow dynamics of cerebral vasculature.

In clinical practice, the acquisition of DSA images during endovascular stroke treatment is commonly performed by manual injection of contrast medium, leading to a high variance of contrast enhancement among DSA images acquired from the same patient at different time points [3]. Adding to this, there is currently a lack of robust tools for automated assessment of cerebral flow and perfusion dynamics. As a result, peri-procedural DSA assessments are primarily assessed visually by interventionists, which is inevitably time-consuming, error-prone, and subjective, particularly considering the high time pressure in some interventional procedures such as endovascular thrombectomy.

With the rapid evolution in AI research, various deep learning-based approaches have been recently proposed for quantitative analysis of DSA [4], such as automated brain perfusion quantification [5, 6], vessel segmentation [7], and vessel occlusion detection [8]. There are an increasing number of DSA analysis methods that incorporate temporal information in AI-based quantification. Nevertheless, the temporal flow dynamics are generally not intuitively visualized and explained in such methods.

While color-coded visualization of quantitative deconvolution-based perfusion parametric imaging in computed tomography (CT) [9] and magnetic resonance imaging (MRI) [10] has been well established, its adoption in DSA is far less widespread. The lack of automatic approaches for reliable assessment of cerebral flow dynamics might be partially due to the projective nature of DSA. In 2010, color-coded DSA was proposed [11, 12]. Since then, it has been used in various clinical scenarios, such as lower extremity intervention [13], intracranial arterial stenosis [14], and pediatric moyamoya disease [15]. However, color-coded DSA is subjective to the contrast injection profile, which hinders cross-patient quantitative analysis. In 2016, Scalzo and Liebeskind [16] demonstrated the feasibility of perfusion parametric angiography. Later Su et al. [17] demonstrated in an animal study that perfusion parametric DSA shows reduced dependency on the contrast injection profile. In the meantime, cerebral perfusion angiography has been demonstrated useful in various studies [18, 19]. However, previous studies are semi-automatic, which requires manual delineations of input arteries. To our knowledge, there exists no fully automatic tool for color-coded quantitative perfusion parametric imaging so far.

The purpose of this study is to develop and validate a framework for fully automated computation of deconvolution-based perfusion parametric images from cerebral DSA. It automatically extracts the arterial input function (AIF) from the supraclinoid segment of the internal carotid artery (ICA) and computes perfusion parametric images, i.e., cerebral blood volume (CBV), cerebral blood flow (CBF), mean transit time (MTT), and Tmax, visualized in a color-coded manner. We hypothesize that these visualizations may reveal insights not directly observable from conventional DSA, potentially enhancing image-guided cerebrovascular interventions and providing novel biomarkers for disease diagnosis and prognosis. Therefore, this work also investigates the association between quantitative perfusion parameters and functional stroke outcomes after endovascular stroke treatment to substantiate the potential value of the proposed framework.

**Fig. 1:**
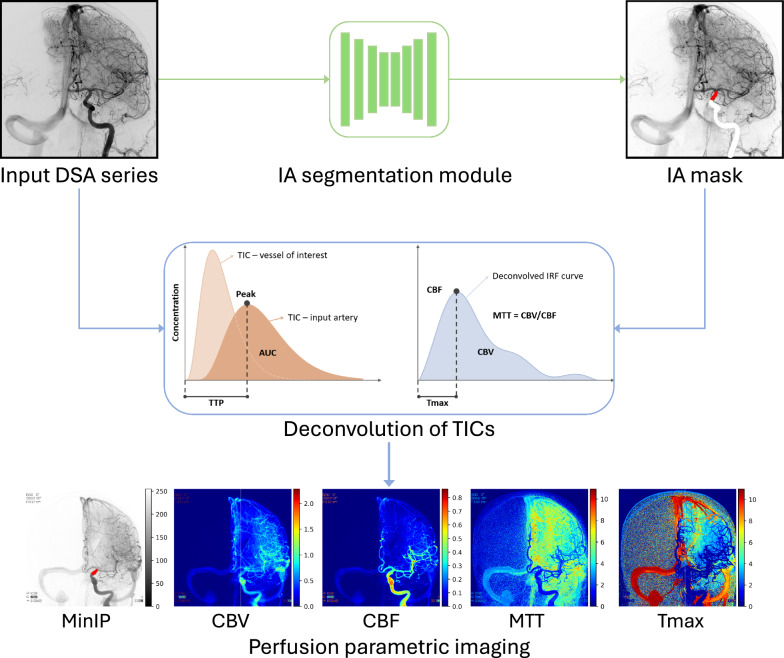
Overview. perfDSA takes a 2D+time DSA series as input and computes the MinIP image across the time axis. The input artery (IA) segmentation module predicts the supraclinoid segment of the internal carotid artery. The deconvolution module takes the input DSA series and the IA mask to compute the perfusion parametric images including CBV, CBF, MTT, and Tmax

## Method

Figure 1 outlines the framework, named perfDSA, for automatic perfusion parametric imaging, comprising two main modules: arterial input function extraction via supraclinoid ICA segmentation, and deconvolution-based parametric image computation. Given an input DSA series, perfDSA first uses a UNet-based network to segment a reference input artery, from where the average AIF is extracted. Subsequently, the AIF is used for deconvolving the time–intensity curves extracted on a pixel-by-pixel basis to generate the impulse residue function (IRF) for each pixel. The DSA perfusion parameters are computed from the IRF per pixel, resulting in various perfusion parametric images.

### Automatic arterial input function extraction

Deconvolution-based perfusion parametric imaging requires an artery input function extracted from an input artery. In this study, we choose the supraclinoid ICA instead of the complete ICA as the reference input artery, based on several previous studies [20–22], which is defined in the Bouthillier classification as C6-C7 segments (clinoid to ICA-terminus) of the internal carotid artery [23]. This choice is motivated by (1) ease of identification, (2) reduced probability of erroneous AIF that may be caused by over-projected contrast material compared to the lower part of the ICA, and (3) reduced errors in the AIF due to stasis of contrast in the lower part of the ICA after injection.

We design a UNet-based vessel segmentation module to automatically segment the reference input artery (IA). We compute the AIF as the average TIC across all TICs from the segmented IA pixels.

**Training** We adopted the well-known UNet architecture. As suggested in [24], we define the loss function $\mathcal {L}_{IAseg}(p, g)$ of this module as a combination of the Dice loss [25] and the cross-entropy loss between the manually delineated supraclinoid ICA and the predicted segmentation:

1 $$\begin{aligned} \mathcal {L}_{IAseg}(p, g)&= \mathcal {L}_{\textrm{DSC}}(p, g) + \mathcal {L}_{\textrm{CE}}(p, g), \end{aligned}$$

where *p* and *g* denote the predicted input artery probability and the reference label, respectively.

**Architecture** The IA segmentation module comprises a contracting encoder path and an expanding decoder path, connected through skip connections. The encoder consists of eight convolutional layers with max-pooling, with the number of channels progressively increasing from 64 to 512. Each convolution uses a 3$\times$3 kernel with a stride of 1. The decoder mirrors this structure by employing eight upsampling layers with 3$\times$3 convolutions and concatenation operations to restore the spatial dimensions to those of the input. Every convolutional layer is accompanied by instance normalization and a ReLU activation function. The final convolutional layer applies a sigmoid activation function, producing pixel-wise probability outputs in the range [0, 1], which are then binarized using a threshold of 0.5.

### Deconvolution-based perfusion parametric imaging

Perfusion parameters, including cerebral blood volume (CBV), cerebral blood flow (CBF), mean transit time (MTT), and Tmax, are extensively used to characterize the microcirculatory properties of tissues and (micro-)vessels. To derive these parametric images from DSA, we adopt the bolus-tracking method similar to those employed in MR and CT angiography. This approach involves deconvolving time–intensity curves (TICs) using the extracted AIF. The deconvolution-based method is based on the indicator dilution theory [26], which states that the contrast concentration at the volume of interest at a time point *t*, $C_{\textrm{voi}}(t)$, is proportional to the product of cerebral blood flow, the mean contrast intensity $\rho _{\textrm{voi}}$ within the volume of interest, and the convolution of the arterial contrast concentration $C_{\textrm{art}}(t)$ with a residue function *r*(*t*):

2 $$\begin{aligned} C_{\textrm{voi}}(t) = \textrm{CBF} \cdot \rho _{\textrm{voi}} \cdot \left( C_{\textrm{art}} * r \right) (t). \end{aligned}$$

For detailed derivations of this formulation, refer to prior studies [27, 28]. Based on this, CBF can be computed as

3 $$\begin{aligned} \textrm{CBF} = \frac{1}{\rho _{\textrm{voi}}} \cdot \frac{C_{\textrm{voi}}(t)}{\left( C_{\textrm{art}} * r \right) (t)}, \end{aligned}$$

where $\rho _{\textrm{voi}}$, $C_{\textrm{voi}}(t)$, and $C_{\textrm{art}}(t)$ are known from DSA series. The maximum of *r*(*t*) is 1 [28], and the time point of this maximum is known as Tmax, expressed as

4 $$\begin{aligned} \textrm{Tmax} = \textrm{argmax}\{t: r(t) = 1\}. \end{aligned}$$

When $C_{\textrm{voi}}(t)$ is deconvolved with $C_{\textrm{art}}(t)$, CBF and *r*(*t*) can be derived as

5 $$\begin{aligned} \textrm{CBF}  &   = \frac{1}{\rho _{\textrm{voi}}} \cdot \frac{C_{\textrm{voi}}(t)}{C_{\textrm{art}}(t)} \quad \text {where} \quad t = \textrm{Tmax}, \quad r(t) \nonumber \\  &   = \frac{1}{\textrm{CBF} \cdot \rho _{\textrm{voi}}} \cdot \frac{C_{\textrm{voi}}(t)}{C_{\textrm{art}}(t)}. \end{aligned}$$

Subsequently, CBV is calculated as the relative blood volume in the volume of interest $C_{\textrm{voi}}$, normalized by the input blood volume $C_{\textrm{art}}$:

6 $$\begin{aligned} \textrm{CBV} = \frac{\int _{0}^{\infty } C_{\textrm{voi}}(\tau ) d\tau }{\int _{0}^{\infty } C_{\textrm{art}}(\tau ) d\tau } = \textrm{CBF} \cdot \rho _{\textrm{voi}} \cdot \int \limits _{0}^{\infty } r(\tau ) d\tau . \end{aligned}$$

Finally, the mean transit time (MTT) is computed using the central volume theorem [29, 30]:

7 $$\begin{aligned} \textrm{MTT} = \frac{\textrm{CBV}}{\textrm{CBF}}. \end{aligned}$$

Following the IA segmentation module, the AIF is extracted as the average TIC of the supraclinoid ICA vessel. Based on the above equations, we automatically obtain the perfusion parametric images.

## Experiments and results

We assess perfDSA in terms of IA segmentation accuracy, and AIF curve similarity compared to a manual approach. Furthermore, we assess the clinical utility of perfDSA in distinguishing good and poor functional recovery in stroke patients with successful endovascular thrombectomy.

### Experimental setup

**Data** This study utilizes a DSA dataset from the MR CLEAN Registry [31], a nationwide multicenter observational cohort in the Netherlands. The registry includes patients who experienced acute ischemic stroke and were treated with endovascular thrombectomy between March 2014 and December 2018. We selected 1006 patients (1882 DSA series) with large vessel occlusions in the internal carotid artery (ICA), supraclinoid ICA, or middle cerebral artery (M1 and M2) segments who achieved successful reperfusion (TICI $\ge$ 2B), as confirmed by a core laboratory (see Supplemental Fig. A1 for the data selection process). The series are acquired in anterior–posterior (AP) or lateral views using various imaging systems (e.g., Philips, GE, Siemens). The DSA series have initial temporal resolutions between 2 and 4 frames per second with series lengths ranging from 10 to 50 frames. An experienced medical doctor created the annotations of the supraclinoid segment of the ICA vessels in the whole dataset, with the assistance of an in-house developed tool in MeVisLab [32]. These annotations serve as the reference standard in this work.

**Implementation details** The framework was implemented in PyTorch, all trained on an NVIDIA RTX A40 GPU. As pre-processing, we resized all frames to $1024\times 1024$ pixels and normalized the intensity values to [0, 1]. The corresponding mask images were also resized to the same resolution. In addition, data augmentation techniques, i.e., horizontal flipping, translation $\in [-5\%, 5\%]$ range, scaling $\in [-5\%, 5\%]$ range, and rotation $\in [-10^{\circ }, 10^{\circ }]$ range, were randomly applied during training with a probability of 0.5 for each. For perfusion parameter computation, we linearly resampled each DSA series to its starting temporal resolution.

We randomly split the dataset into training, validation, and testing sets with a ratio of 50%-20%-30% on the patient level, stratified on the dichotomized modified Rankin Scale (mRS) score, a categorical measure of functional independence in stroke. This resulted in 503 patients (949 DSA series) for training, 201 (373) for validation, and 302 (560) for testing. The model was trained using RMSprop optimization and a ReduceLROnPlateau scheduler with a patience of 10 epochs, a decay factor of 0.5, and an initial learning rate of $1 \times 10^{-6}$. An early stopping strategy was applied with a patience of 50 epochs and a maximum of 1000 epochs.

**Fig. 2:**
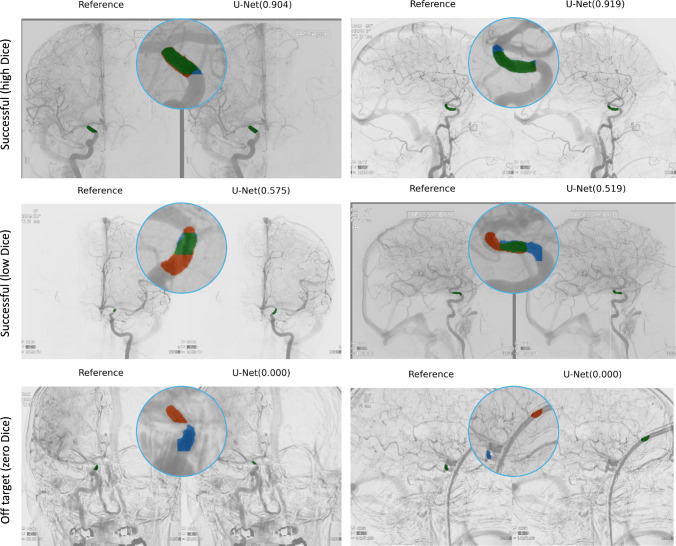
Selected examples of input artery segmentation. The circle area above each example shows the segmentation error map, where blue represents false negatives, red indicates false positives, and green refers to true positives. In brackets are the corresponding Dice scores obtained using the UNet-based approach

### Results

**Input artery segmentation** In Table 1, we present the performance of perfDSA for input artery segmentation. The proposed perfDSA framework achieved an overall Dice of 0.73 (±0.21), which is considered good given the rather small target vessel object and high boundary uncertainties of those manual annotations. The model achieved nearly 100% specificity with a sensitivity of 79%, indicating that it tends to under-segment the target vessel object. Based on a visual assessment, the on-target rate (i.e., Dice > 0) of input artery segmentation was 98%.

**Fig. 3:**
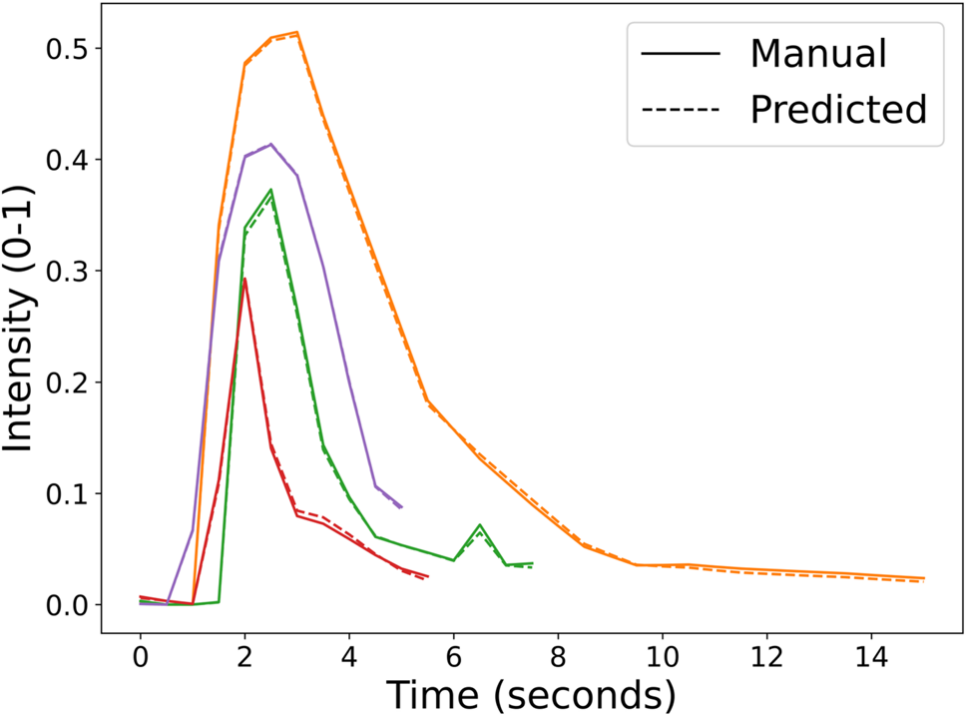
Example AIFs extracted using perfDSA (dashed lines) versus those from manually annotated masks (solid lines)

In Fig. 2, we present six example visualizations of segmentation results in AP and lateral view. The top row shows excellent examples with high Dice, demonstrating the high accuracy of the segmentation module. The middle row includes two examples where the predicted segmentations are well on target despite relatively low Dice coefficients. This is largely due to high uncertainties on the segment boundaries. The bottom row showcased off-target examples where the module finds difficulty in identifying the exact segment, likely due to motion artifacts or static instruments.

**Table 1:** Performance of perfDSA in input artery segmentation and AIF extraction

Method	Input artery segmentation				AIF extraction	
	On-target rate	Dice	Spec	Sens	RMSE	PCC
perfDSA	0.98	0.73$\,\pm \, 0.21$	$1 \,\pm \, 0$	$0.79 \,\pm \, 0.22$	$0.01 \,\pm \, 0.02$	$0.99 \,\pm \, 0.09$

**Arterial input function** To better understand what a Dice of 0.73 means to the resulting AIF curves, we compared the AIF curves extracted from the IA segmentation module of perfDSA with those from the manual IA masks. We obtained a high Pearson correlation coefficient (PCC) of 0.99 and a root mean squared error (RMSE) of 0.01, corresponding to 2.55 gray levels out of 255. It is also shown in Fig. 3 that the resulting AIFs are nearly identical to those from the manually annotated ones.

**Fig. 4:**
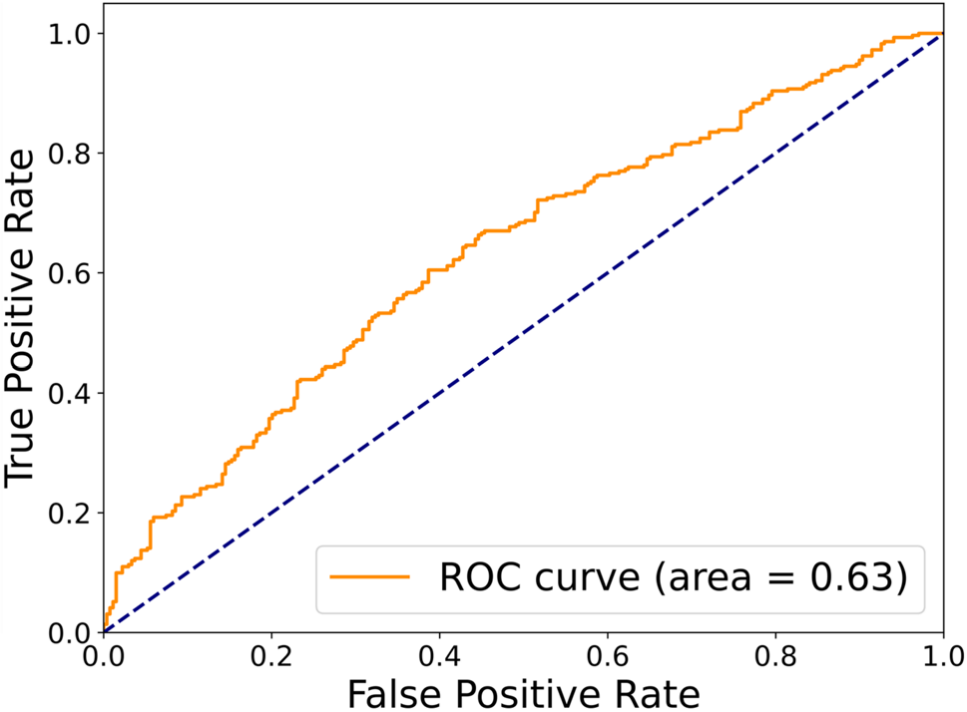
ROC curve of logistic regression for stroke functional outcome prediction using perfusion parameters from perfDSA

**Runtime** For each DSA series, the proposed perfDSA takes on average 0.004 s to automatically extract the AIF on an NVIDIA 2080 Ti GPU and approximately 1 s to generate all deconvolution-based perfusion parametric images on an AMD Ryzen Threadripper 1920X 12-core CPU (Fig. 4). This promises timely treatment assessment and decision support in image-guided endovascular interventions in clinical practice.

**Fig. 5:**
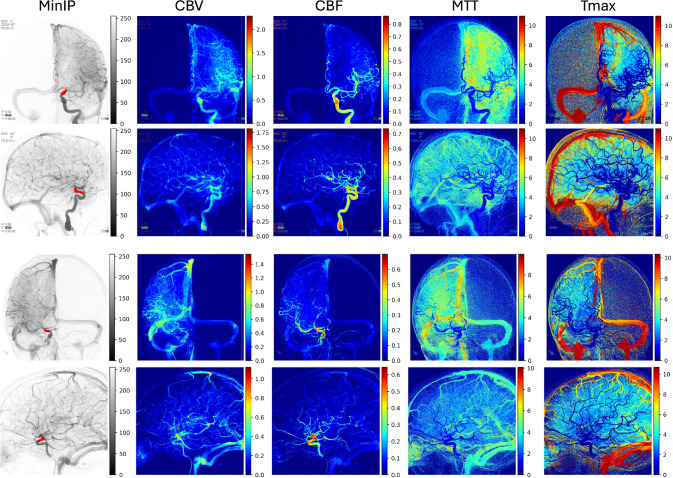
Examples of deconvolution-based perfusion parametric images produced by perfDSA. From left to right: MinIP image of the DSA, CBV, CBF, MTT, and Tmax

**Perfusion parametric imaging** In Fig. 5, we present several examples of deconvolution-based perfusion parametric images produced by perfDSA. These color-coded images visualize various aspects of the temporal flow and perfusion dynamics in 2D images. If certain anatomical regions are of interest, average perfusion parameters could be easily computed from given region masks, such as internal carotid artery (ICA) territory, middle cerebral artery (MCA) territory, or the motor cortex.

**Application: quantitative perfusion angiography associates with stroke functional outcome** The proposed perfDSA provides fully automatic quantitative perfusion parameter image generation, which may reveal insights that are not directly observed in DSA. In clinical practice, this functionality would assist therapeutic guidance in cerebrovascular interventions, and facilitate cross-patient large-scale quantitative analyses. In this proof-of-concept experiment, we collected DSA images of stroke patients who had successful endovascular thrombectomy. However, nearly half of the patients did not recover to functional independence. We investigated whether perfDSA could provide additional insights into this phenomenon.

From each of the 560 DSA images in the test set, we extracted the average CBV, CBF, MTT, and Tmax from the ICA and MCA territories, respectively, resulting in eight parameters per image. We performed logistic regression to predict the dichotomized functional outcome associated with each image, measured by the dichotomized mRS score. perfDSA was able to distinguish favorable functional outcomes with an area under the ROC curve (AUC) of 0.63 (Fig. 4). As shown in Table 2, the Wald test reveals that CBF and Tmax are both significantly ($P<0.05$) associated with favorable functional outcomes. These interesting initial findings necessitate further large-scale clinical validations.

**Table 2:** Association between perfusion parameters and stroke functional outcome

Perfusion parameters		ICA territory				MCA territory				Overall
		CBV	CBF	MTT	Tmax	CBV	CBF	MTT	Tmax	
Odds ratio	Mean	1.34	0.04	1.49	0.24	1.06	22.27	0.45	4.54	n/a
	Std	± 1.95	± 0.05	± 1.62	± 0.12	± 1.46	± 27.19	± 0.49	± 2.24	
*P*-value		0.84	**0**.**01**	0.72	**3.54e** $-$ **3**	0.97	**0**.**01**	0.47	**2.19e** $-$ **3**	**2.62e** $-$ **5**

The bold P-values ($P < 0.05$) indicate statistical significance

## Discussion and conclusion

We introduced perfDSA, an automated approach for perfusion parametric imaging in DSA. This method utilizes a UNet-based module to segment the supraclinoid internal carotid artery, serving as the reference input artery, and generates perfusion parametric images through a deconvolution-based bolus-tracking technique.

The proposed UNet-based AIF extraction module marks an initial exploration of deep learning-based segmentation of the supraclinoid internal carotid artery, achieving a Dice coefficient of 0.73. While this value may seem moderate, qualitative assessments confirm high-quality segmentation results. Considering the small size of the target segment (Fig. 2) and the inherent boundary uncertainties in manual annotations, the presented performance is rather competitive. As the annotations were created by a single individual, inter-annotator variability was not quantitatively assessed. The nearly 100% segmentation specificity indicates an almost negligible false positive rate, which is critical for robust and accurate AIF extraction. In the subsequent quantitative analysis, the competitive performance is also evident from the high similarity (PCC=0.99, Table 1) between the automated and manually extracted AIFs. Alternative segmentation models can be explored within the modular design of perfDSA. Examples include nnUNet [24], a leading benchmark in medical image segmentation tasks, and CAVE [7], a specifically tailored model for vessel segmentation in DSA. While these models may obtain slightly better segmentation accuracy, the room for improvement in resulted AIF similarity is likely limited. Nevertheless, it is valuable as future work to enhance perfDSA with state-of-the-art segmentation models.

The framework enables the automatic generation of deconvolution-based perfusion parametric DSA images. While these parameters demonstrate reduced dependency on the contrast injection profile, current evidence does not confirm their full quantitative accuracy. Future work should focus on validating the relationship between parameters derived from DSA and those obtained from magnetic resonance angiography (MRA) within the same patient cohort.

Experimental results on a subset of the MR CLEAN Registry highlight the potential of perfDSA to extract image-based insights beyond what is directly identifiable through qualitative analysis. This capability promises advancements in various downstream quantitative cerebral analysis tasks. Subsequent research should explore these insights in large-scale clinical studies to thoroughly assess these initial findings.

In conclusion, perfDSA provides the community with a tool for automatically quantifying cerebral perfusion in DSA. It has the potential to inform new strategies for improving image-guided diagnosis and cerebral interventions.

## Supplementary Information

Below is the link to the electronic supplementary material.

**Supplementary information** This article has accompanying supplementary materials. (pdf 193KB)

## Data Availability

The source code is available at https://github.com/RuishengSu/perfDSA.
