# Supplementary material for: perfDSA: Automatic Perfusion Imaging in Cerebral Digital Subtraction Angiography
Source: Int J Comput Assist Radiol Surg. 2025 Apr 24;20(6):1195–203. doi: 10.1007/s11548-025-03359-4 (PMC12167352; doi:10.1007/s11548-025-03359-4)
Supplement: Supplementary file 1 — Supplementary information This article has accompanying supplementary materials. (pdf 193KB) [file 11548_2025_3359_MOESM1_ESM.pdf]

Appendix A

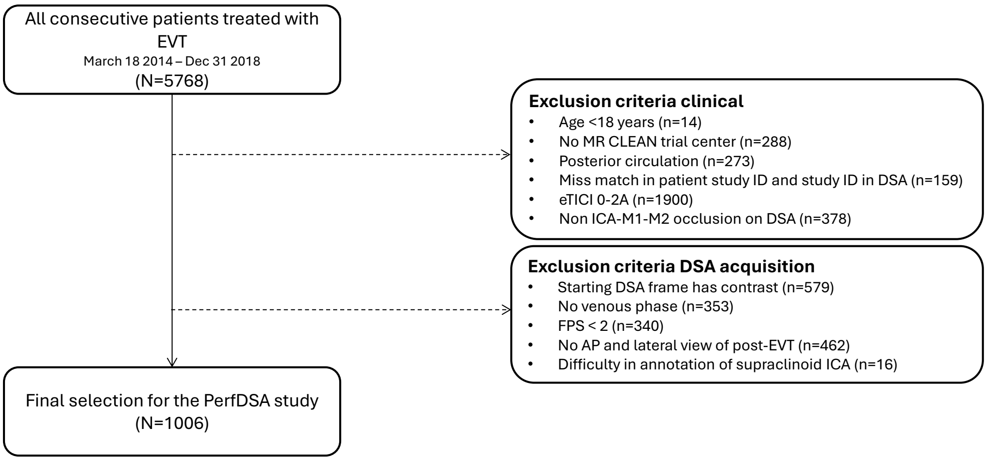

Fig. A1: Detailed DSA data selection flow diagram for this study.

Appendix B

Disclosures

Erasmus MC received funds from Stryker® by DD, AvdL, and Bracco Imaging® by DD. Amsterdam UMC received funds from Stryker® for consultations by CM, YR, and OB. MUMC received funds from Stryker® and Codman® for consultations by WZ.

MR CLEAN Registry Investigators – group authors

Executive committee

Diederik W.J. Dippel[1]; Aad van der Lugt[2]; Charles B.L.M. Majoie[3]; Yvo B.W.E.M. Roos[4]; Robert J. van Oostenbrugge[5,41]; Wim H. van Zwam[6,41]; Jelis Boiten[14]; Jan Albert Vos[8]

Study coordinators

Ivo G.H. Jansen[3]; Maxim J.H.L. Mulder[1], 2; Robert-Jan B. Goldhoorn[5,6,41]; Kars C.J. Compagne[2]; Manon Kappelhof[3]; Josje Brouwer[4]; Sanne J. den Hartog[1,2,40]; Wouter H. Hinsenveld[5,6]

Local principal investigators

Diederik W.J. Dippel[1]; Bob Roozenbeek[1]; Aad van der Lugt[2]; Adriaan C.G.M. van Es[2]; Charles B.L.M. Majoie[3]; Yvo B.W.E.M. Roos[4]; Bart J. Emmer[3]; Jonathan M. Coutinho[4]; Wouter J. Schonewille[7]; Jan Albert Vos[8]; Marieke J.H. Wermer[9]; Marianne A.A. van Walderveen[10]; Julie Staals[5,41]; Robert J. van Oostenbrugge[5,41]; Wim

H. van Zwam[6,41]; Jeannette Hofmeijer[11]; Jasper M. Martens[12]; Geert J. Lycklama à Nijeholt[13]; Jelis Boiten[14]; Sebastiaan F. de Bruijn[15]; Lukas C. van Dijk[16]; H. Bart van der Worp[17]; Rob H. Lo[18]; Ewoud J. van Dijk[19]; Hieronymus D. Boogaarts[20]; J. de Vries[22]; Paul L.M. de Kort[21]; Julia van Tuijl[21]; Jo P. Peluso[26]; Puck Fransen[22]; Jan S.P. van den Berg[22]; Boudewijn A.A.M. van Hasselt[23]; Leo A.M. Aerden[24]; René J. Dallinga[25]; Maarten Uyttenboogaart[28]; Omid Eschgi[29]; Reinoud P.H. Bokkers[29]; Tobien H.C.M.L. Schreuder[30]; Roel J.J. Heijboer[31]; Koos Keizer[32]; Lonneke S.F. Yo[33]; Heleen M. den Hertog[22]; Tomas Bulut[35]; Paul J.A.M. Brouwers[34]

Imaging assessment committee
Charles B.L.M. Majoie[3] (chair); Wim H. van Zwam[6,41]; Aad van der Lugt[2]; Geert J. Lycklama à Nijeholt[13]; Marianne A.A. van Walderveen[10]; Marieke E.S. Sprengers[3]; Sjoerd F.M. Jenniskens[27]; René van den Berg[3]; Albert J. Yoo[38]; Ludo F.M. Beenen[3]; Alida A. Postma[6,42]; Stefan D. Roosendaal[3]; Bas F.W. van der Kallen[13]; Ido R. van den Wijngaard[13]; Adriaan C.G.M. van Es[2]; Bart J. Emmer[3]; Jasper M. Martens[12]; Lonneke S.F. Yo[33]; Jan Albert Vos[8]; Joost Bot[36]; Pieter-Jan van Doormaal[2]; Anton Meijer[27]; Elyas Ghariq[13]; Reinoud P.H. Bokkers[29]; Marc P. van Proosdij[37]; G. Menno Krietemeijer[33]; Jo P. Peluso[26]; Hieronymus D. Boogaarts[20]; Rob Lo[18]; Wouter Dinkelaar[2]; Auke P.A. Appelman[29]; Bas Hammer[16]; Sjoert Pegge[27]; Anouk van der Hoorn[29]; Saman Vinke[20]

Writing committee
Diederik W.J. Dippel[1] (chair); Aad van der Lugt[2]; Charles B.L.M. Majoie[3]; Yvo B.W.E.M. Roos[4]; Robert J. van Oostenbrugge[5,41]; Wim H. van Zwam[6,41]; Geert J. Lycklama à Nijeholt[13]; Jelis Boiten[14]; Jan Albert Vos[8]; Wouter J. Schonewille[7]; Jeannette Hofmeijer[11]; Jasper M. Mart

Adverse event committee
Robert J. van Oostenbrugge[5,41](chair); Jeannette Hofmeijer[11]; H. Zwenneke Flach[23].

Trial methodologist
Hester F. Lingsma[40].

Research nurses / local trial coordinators
Naziha el Ghannouti[1]; Martin Sterrenberg[1]; Wilma Pellikaan[7]; Rita Sprengers[4]; Marjan Elfrink[11]; Michelle Simons[11]; Marjolein Vossers[12]; Joke de Meris[14]; Tamara Vermeulen[14]; Annet Geerlings[19]; Gina van Vemde[22]; Tiny Simons[30]; Gert Messchendorp[28]; Nynke Nicolaij[28]; Hester Bongenaar[32]; Karin Bodde[24]; Sandra Kleijn[34]; Jasmijn Lodico[34]; Hanneke Droste[34]; Maureen Wollaert[5]; Sabrina Verheesen[5]; D. Jeurrissen[5]; Erna Bos[9]; Yvonne Drabbe[15]; Michelle Sandiman[15]; Nicoline Aaldering[11]; Berber Zweedijk[17]; Jocova Vervoort[21]; Eva Ponjee[22]; Sharon Romviel[19]; Karin Kanselaar[19]; Denn Barning[10].

PhD / Medical students

Esmee Venema[40]; Vicky Chalos[1,40]; Ralph R. Geuskens[3]; Tim van Straaten[19]; Saliha Ergezen[1]; Roger R.M. Harmsma[1]; Daan Muijres[1]; Anouk de Jong[1]; Olvert A. Berkhemer[1,3,6]; Anna M.M. Boers[3,39]; J. Huguet[3]; P.F.C. Groot[3]; Marieke A. Mens[3]; Katinka R. van Kranendonk[3]; Kilian M. Treurniet[3]; Manon L. Tolhuisen[3,39]; Heitor Alves[3]; Annick J. Weterings[3]; Eleonora L.F. Kirkels[3]; Eva J.H.F. Voogd[11]; Lieve M. Schupp[3]; Sabine L. Collette[28,29]; Adrien E.D. Groot[4]; Natalie E. LeCouffe[4]; Praneeta R. Konduri[39]; Haryadi Prasetya[39]; Nerea Arrarte-Terreros[39]; Lucas A. Ramos[39].

List of affiliations

Department of Neurology[1], Radiology[2], Public Health[40], Erasmus MC University Medical Center;
Department of Radiology and Nuclear Medicine[3], Neurology[4], Biomedical Engineering & Physics[39], Amsterdam UMC, location University of Amsterdam;
Department of Neurology[5], Radiology & Nuclear Medicine[6], Maastricht University Medical Center; School for Cardiovascular Diseases Maastricht (CARIM)[41]; and MHeNs School for Mental Health and Neuroscience, Maastricht, the Netherlands[42];
Department of Neurology[7], Radiology[8], Sint Antonius Hospital, Nieuwegein;
Department of Neurology[9], Radiology[10], Leiden University Medical Center;
Department of Neurology[11], Radiology[12], Rijnstate Hospital, Arnhem;
Department of Radiology[13], Neurology[14], Haaglanden MC, the Hague;
Department of Neurology[15], Radiology[16], HAGA Hospital, the Hague;
Department of Neurology[17], Radiology[18], University Medical Center Utrecht;
Department of Neurology[19], Neurosurgery[20], Radiology[27], Radboud University Medical Center, Nijmegen;
Department of Neurology[21], Radiology[26], Elisabeth-TweeSteden ziekenhuis, Tilburg;
Department of Neurology[22], Radiology[23], Isala Klinieken, Zwolle;
Department of Neurology[24], Radiology[25], Reinier de Graaf Gasthuis, Delft;
Department of Neurology[28], Radiology[29], University Medical Center Groningen;
Department of Neurology[30], Radiology[31], Atrium Medical Center, Heerlen;
Department of Neurology[32], Radiology[33], Catharina Hospital, Eindhoven;
Department of Neurology[34], Radiology[35], Medisch Spectrum Twente, Enschede;
Department of Radiology[36], Amsterdam UMC, Vrije Universiteit van Amsterdam, Amsterdam;
Department of Radiology[37], Noordwest Ziekenhuisgroep, Alkmaar;
Department of Radiology[38], Texas Stroke Institute, Texas, United States of America.
